# Fever Screening at Airports and Imported Dengue

**DOI:** 10.3201/eid1103.040420

**Published:** 2005-03

**Authors:** Pei-Yun Shu, Li-Jung Chien, Shu-Fen Chang, Chien-Ling Su, Yu-Chung Kuo, Tsai-Ling Liao, Mei-Shang Ho, Ting-Hsiang Lin, Jyh-Hsiung Huang

**Affiliations:** * Center for Disease Control, Taipei, Taiwan, Republic of China; † Institute of Biomedical Science, Taipei, Taiwan, Republic of China

## Abstract

Airport fever screening in Taiwan, July 2003–June 2004, identified 40 confirmed dengue cases. Results obtained by capture immunoglobulin (Ig) M and IgG enzyme-linked immunoassay, real time 1-step polymerase chain reaction, and virus isolation showed that 33 (82.5%) of 40 patients were viremic. Airport fever screening can thus quickly identify imported dengue cases.

Dengue viruses are arboviruses that cause substantial human disease in tropical and subtropical regions of the world, especially in urban and semiurban areas. Because of its high endemicity in many countries in the Western Pacific, Southeast Asia, and South American regions, dengue has become an important public health problem in most nations in these areas (*1*). Dengue is not considered endemic in Taiwan, however, and the constant importation of dengue viruses from the neighboring Southeast Asian countries through close commercial links and air travel is believed to cause local outbreaks (*2**,**3*). Until now, local outbreaks, which are most frequent in the summer and fall, have each been caused by a single imported dengue virus strain that disappears when each outbreak ends. Because waves of relatively cold temperature of ≈10°C cause low mosquito density in winter, winter outbreaks are rare, with the exceptions of large outbreaks in 1915–1916, 1942–1943, 1987–1988, and 2001–2002. Outbreaks occur mainly in southern Taiwan, where *Aedes aegypti* and *A*. *albopictus* coexist, and rarely occur in central and northern Taiwan, where only *A*. *albopictus* exist. The dengue hemorrhagic fever cases in Taiwan are highly correlated with increasing age and secondary dengue virus infection (*4*; J-H Huang, unpub data).

## The Study

To identify imported dengue cases and reduce the local spread of newly introduced dengue viruses, the health authority, now Center for Disease Control, Taiwan, has established an integrated dengue control program that includes various surveillance systems, a network of rapid diagnostic laboratories, and mechanisms of rapid response to implement control measures (*3*). The primary objective is to prevent the introduction of new dengue viruses into Taiwan by travel and subsequent local spread. Dengue is classified as a reportable infectious disease, and suspected cases must be reported within 24 hours of clinical diagnosis in Taiwan. For effective surveillance, both passive (hospital-based reporting system) and active (such as health statement of inbound passengers, self-report, expanded screening for contacts of confirmed cases, patients with fever of unknown origin, school-based reporting, community screening) surveillance systems were established in central and local health departments.

For rapid diagnosis, 2 central dengue diagnostic laboratories were set up in Center for Disease Control–Taiwan, at Kun-Yan Laboratory in northern Taiwan and at a fourth branch laboratory in southern Taiwan. Serum samples from suspected dengue patients were sent to the diagnostic laboratories, and the results were reported within 24 to 48 hours. To avoid delays, the laboratory is scheduled to perform the tests on a daily basis without vacations. A rapid diagnostic system was developed to detect and differentiate various flavivirus infections on the basis of the results of 1-step real-time polymerase chain reaction (PCR) and envelope membrane (E/M)–specific capture immunoglobulin (Ig) M and IgG enzyme-linked immunosorbent assay (ELISA) (*5**–**7*). Analysis of a total of 959 acute- and convalescent-phase serum specimens from 799 confirmed dengue patients showed that 95% of acute-phase serum specimens could be identified as being from confirmed or probable case-patients based on these 2 assays (*8*).

In 2003, Taiwan was one of the countries heavily affected by the multinational epidemics of severe acute respiratory syndrome (SARS) (*9*). During the SARS epidemic, the body temperature of all inbound and outbound passengers at the 2 international airports was screened to prevent international spread of SARS. Since July 14, 2003, all inbound passengers have been required to complete the “SARS Survey Form” before landing and to have their body temperature taken by an infrared thermal camera. Any passenger showing body temperature >37°C is rechecked by ear temperature, and serum samples are collected and sent for SARS diagnosis if the ear temperature is >37.5°C. After July 5, 2003, the world was largely considered to be SARS free, and other causes of fever had to be considered; therefore, a panel of diagnostic tests, including tests for pathogens of dengue, malaria, enteric bacteria, and other diseases (such as yellow fever, plague), was performed for selected fever patients. Since dengue fever is among the top yearly imported reportable diseases in Taiwan, we began a trial fever screening program for dengue along with SARS screening at the airports.

We report our findings on early identification of dengue fever through fever screening at the 2 international airports, C.K.S. and Kaohsiung Airports, Taiwan. More than 8,000,000 inbound travelers passed through the 2 airports from July 2003 to June 2004. Among these, ≈22,000 passengers were identified as fever patients by an infrared thermal camera and rechecked by ear temperature. Diagnostic testing algorithms for screened fever patients were based on evaluation by airport clinicians. After clinical diagnosis, 3,011 serum samples were sent for laboratory diagnosis of dengue virus infection. Forty (1.33%) of 3,011 serum samples were confirmed to be positive on the basis of the results of real-time PCR and E/M-specific capture IgM and IgG ELISA. During the same period, 6,005 dengue cases were reported in Taiwan (both indigenous and imported cases), which includes 935 cases from the passive surveillance system and 5,070 cases from active surveillance systems (3,011 fever patients were identified by fever screening and 2,059 cases were identified from other systems). Among these, 73 were confirmed to be imported dengue cases, including 25 cases reported from hospitals through passive surveillance and 48 cases identified by active surveillance, such as airport screening and self-report by patients. Airport fever screening alone picked up 40 (83.3%) of 48 of all imported cases identified by the active surveillance system. Thus, 8 imported cases were identified by other active surveillance methods. The average length between the onsets of dengue symptoms to the time of diagnosis was 4.15 days for the 40 case-patients who were identified at the airport, as opposed to 11 days for those who were reported from the hospital. Whether the shorter length required to diagnose conditions identified by airport fever screening contributed to the low indigenous dengue in the season warrants further investigation.

Fever screening at the airports has also dramatically increased the proportion of imported dengue cases identified by active surveillance, 48 (65.8%), of 73 which is significantly higher than the number identified during years before fever screening were implemented (p < 0.0001 by chi-square test) (Table 1). The countries of origin of imported dengue fever from July 2003 to June 2004 were all located in the Western Pacific and Southeast Asia (Table 2). The distribution of the countries of origin accurately reflected the frequency of air travel between Taiwan and these nations, as well as the intensity of massive dengue outbreaks during the same period in the country of origin. Analyses of dengue virus serotypes showed that various serotypes were circulating in each of these countries during this period.

**Table 1:** Summary of imported dengue cases identified by passive and active surveillance systems in Taiwan from 1998 to June 2004

Year	Total	Passive surveillance, no. cases (%)	Active surveillance, no. cases (%)
1998	110	96 (87.3)	14 (12.7)
1999	29	24 (82.8)	5 (17.2)
2000	27	23 (85.2)	4 (14.8)
2001	56	46 (82.1)	10 (17.9)
2002	52	42 (80.8)	10 (19.2)
Jan 2003–June 2003	20	18 (90.0)	2 (10.0)
Jul 2003–June 2004	73	25 (34.2)	48 (65.8)

**Table 2:** Countries of origin and dengue virus serotype of imported dengue cases in Taiwan, July 2003–June 2004

Country of origin	No. imported cases		Serotype*				
	Total	Fever screening	D-1	D-2	D-3	D-4	Unknown
Vietnam	21	13	0	11	3	3	4
Indonesia	15	8	3	6	2	1	3
The Philippines	15	7	5	2	0	5	3
Thailand	11	7	3	3	1	3	1
India	3	1	0	2	0	0	1
Malaysia	2	2	0	2	0	0	0
Myanmar	1	0	0	0	0	0	1
Cambodia	4	2	1	2	0	0	1
Sri Lanka	1	0	0	0	0	0	1
Total	73	40	12	28	6	12	15

*Dengue virus serotypes were identified by real time 1-step polymerase chain reaction, virus isolation, or both, for all imported cases.

Most of the confirmed cases (33 of 40) identified by airport fever screening were viremic (real-time PCR positive, IgM and IgG negative). The other 7 case-patients tested positive for dengue-specific IgM or IgG antibody, although they were febrile at the time of testing (data not shown). Estimating how many patients might have been viremic but were not picked up by the system is difficult, since persons infected with dengue virus are usually viremic from 2 to 3 days before onset of symptoms until defervescence.

## Conclusions

Our results demonstrated that fever screening at airports is an effective means of identifying imported dengue cases, whereas the health statements of inbound passengers, which have been required for years, are ineffective. Although fever screening with infrared temperature screening was implemented in an attempt to avoid SARS transmission, it proved to be effective in active surveillance of dengue. This approach seems promising for dengue and perhaps for other diseases and should be further evaluated.

The cost of identifying dengue virus infections with airport fever screening is similar to that of other surveillance methods. The airport fever screening method requires an infrared thermal camera, which costs approximately U.S. $43,000 for each set of instruments. In addition, 1 additional worker is needed to monitor this alarm system. The reporting procedure and clinical and laboratory diagnoses are similar to those of surveillance methods. Therefore, the method is a cost-effective means of identifying imported dengue cases.

Although febrile passengers suspected of having dengue virus infection were not detained at the airport, and an epidemiologic investigation was not conducted, they were provided with a mosquito net to avoid mosquito bites and instructed to report to the local health department if they felt ill. Laboratory diagnoses were performed on a daily basis, and results were reported within 24 to 48 hours. Control measures were implemented as soon as possible if probable or confirmed dengue cases were identified. Since viremic persons, going about their normal activities for a mean interval of 2 to 3 days before diagnosis, could have transmitted dengue, the laboratory detection method on its own will not be effective in preventing transmission. Therefore, developing an integrated program that includes various surveillance systems, rapid diagnostic laboratories, and emergency control measures is necessary to prevent the introduction and spread of new dengue viruses into a region. Control measures should consist of epidemiologic investigation, health education, analysis of mosquito density, source reduction, and insecticide application. As part of an integrated dengue control program, fever screening at the airport has become one of the most important active surveillance systems in Taiwan since its introduction in July 2003. We believed that this active surveillance system could also be successfully applied to screen febrile patients and reduce the introduction of many potential infectious diseases.

This work was in part supported by grants DOH92-DC-2005 and DOH92-DC-2006 from the Center for Disease Control, Department of Health, Taipei, Taiwan, Republic of China.

Dr. Shu is a research fellow in the Center for Research and Diagnostics, Center for Disease Control, Department of Health, Taipei, Taiwan, Republic of China. She played a key role in developing the rapid diagnostic system for flavivirus infection. Her research interests include viral hepatitis, flavivirus infections, and rickettsial diseases.
